# Plasma omentin levels are inversely associated with atherosclerosis in type 2 diabetes patients with increased plasma adiponectin levels: a cross-sectional study

**DOI:** 10.1186/s12933-019-0973-3

**Published:** 2019-12-05

**Authors:** Masami Nishimura, Tomoaki Morioka, Mariko Hayashi, Yoshinori Kakutani, Yuko Yamazaki, Masafumi Kurajoh, Katsuhito Mori, Shinya Fukumoto, Atsushi Shioi, Tetsuo Shoji, Masaaki Inaba, Masanori Emoto

**Affiliations:** 1 Department of Metabolism, Endocrinology and Molecular Medicine, Osaka City University Graduate School of Medicine, 1-4-3, Asahi-machi, Abeno-ku, Osaka, 545-8585 Japan; 2 Department of Nephrology, Osaka City University Graduate School of Medicine, 1-4-3, Asahi-machi, Abeno-ku, Osaka, 545-8585 Japan; 3 Department of Premier Preventive Medicine, Osaka City University Graduate School of Medicine, 1-4-3, Asahi-machi, Abeno-ku, Osaka, 545-8585 Japan; 4 Department of Vascular Medicine, Osaka City University Graduate School of Medicine, 1-4-3, Asahi-machi, Abeno-ku, Osaka, 545-8585 Japan; 5 Vascular Science Center for Translational Research, Osaka City University Graduate School of Medicine, 1-4-3, Asahi-machi, Abeno-ku, Osaka, 545-8585 Japan

**Keywords:** Omentin, Adiponectin, Atherosclerosis, Intima-media thickness, Type 2 diabetes

## Abstract

**Background:**

Omentin and adiponectin are among the anti-inflammatory and anti-atherogenic adipokines that have potentially beneficial effects on cardiovascular disorders. Recent studies indicate a paradoxical relationship between adiponectin and cardiovascular mortality across many clinical settings including type 2 diabetes. In this study, we characterized the clinical features of type 2 diabetes patients with increased adiponectin levels and examined the association between omentin and atherosclerosis in those patients.

**Methods:**

The subjects were 413 patients with type 2 diabetes. Fasting plasma omentin and total adiponectin levels were measured by enzyme-linked immunosorbent assay. The intima-media thickness (IMT) of the common carotid artery was measured by ultrasonography. The subjects were stratified according to the median value of plasma adiponectin.

**Results:**

In high-adiponectin group, omentin levels were higher, while IMT tended to be greater than those in low-adiponectin group. The high-adiponectin group also exhibited older age, higher systolic blood pressure, lower kidney function, body mass index, and insulin resistance index compared to the low-adiponectin group. Multivariate analysis revealed that omentin levels were independently and negatively associated with IMT in high-adiponectin group, but not in low-adiponectin group, after adjusting for adiponectin levels and traditional cardiovascular risk factors. On the other hand, adiponectin levels were not significantly associated with IMT in either group.

**Conclusions:**

Plasma omentin levels are inversely associated with IMT in type 2 diabetes patients with increased adiponectin levels and multiple cardiovascular risk factors. This study suggests a protective role of omentin against atherosclerosis in type 2 diabetes patients, which is potentially influenced by adiponectin level and cardiovascular risk status.

## Background

Omentin, also referred as intelectin-1, was first identified as a galactofuranose-binding lectin [1], and is abundantly expressed in human omental adipose tissue [2]. It enhances insulin action in human adipocytes in vitro [2] and its circulating levels are decreased in individuals with obesity [3, 4] and/or patients with type 2 diabetes (T2D) [3, 5, 6]. Omentin also has beneficial effects on cardiovascular system in mice through its actions on endothelial cells, smooth muscle cells, macrophages, and cardiomyocytes [7, 8]. In line with the experimental evidence, decreased circulating omentin levels were observed in patients with coronary artery disease [9], peripheral artery disease [10], and established carotid atherosclerosis [11] compared to those without. An inverse relationship was also observed in recent clinical studies between circulating omentin levels and surrogate makers of cardiovascular risk, including arterial stiffness [6], endothelial dysfunction [12], carotid intima-media thickness (IMT) [13, 14], and carotid plaque instability [15]. More recently, we reported that plasma omentin levels were higher in T2D patients than healthy controls and were positively associated with flow-mediated vasodilatation, a marker of endothelial function, in high-risk subgroups of T2D patients with older age, reduced kidney function, or preexisting cardiovascular diseases (CVDs) [16]. Those prior studies [6, 9–14], including ours [16], suggest a protective role of human omentin on atherosclerotic CVDs.

Adiponectin is well-recognized as an anti-inflammatory adipocytokine which exerts beneficial actions on metabolic and cardiovascular disorders [8, 17, 18]. Previous reports consistently demonstrated that circulating adiponectin levels were decreased in obesity, T2D, and coronary artery disease [8, 17, 18]. However, despite its anti-inflammatory and cardioprotective properties in cellular and animal models, a number of prospective human studies have indicated a positive, rather than the expected negative, association between circulating adiponectin levels and all-cause and cardiovascular mortality across many clinical conditions including diabetes, CVDs, and chronic kidney disease [19, 20]. Regarding subclinical atherosclerosis, conflicting results were reported on the association between hypoadiponectinemia and severity of carotid IMT, especially in subjects with metabolic disorders or inflammatory diseases [21]. Namely, most studies in relatively healthy subjects or subjects with wide range of glucose tolerance significantly associated lower adiponectin levels with greater IMT, while the majority of studies in subjects with T2D alone failed to show a significant association between adiponectin and IMT [21]. These clinical findings collectively suggest that adiponectin fails to exert its protective effect against atherosclerotic CVDs in individuals with T2D.

Both omentin and adiponectin are among the adipokines with anti-inflammatory and cardioprotective effects in experimental models [8]. However, no prior study has investigated a direct molecular link between omentin and adiponectin. Evidence from clinical studies indicates that circulating omentin levels are increased, while adiponectin levels are decreased in proinflammatory states such as type 1 diabetes, Crohn’s disease, and nonalcoholic fatty liver disease [7]. The reciprocal regulation observed between omentin and adiponectin may point towards a more important role of omentin as a regulator of inflammatory process. In addition, our recent study showed that omentin, but not adiponectin, was positively associated with vascular endothelial function in high CVD risk subgroups of T2D patients [16], suggesting a dominant role of omentin, rather than adiponectin, against endothelial dysfunction in patients at high CVD risk status. In the present study, we hypothesized that omentin plays an anti-atherogenic effect, while adiponectin levels are increased but lose its effect, against atherosclerosis in patients with high-risk status for CVDs. To this end, we investigated the clinical features of patients with T2D and increased plasma adiponectin levels, and the relationship between plasma omentin levels and carotid IMT in those patients.

## Methods

### Study design and subjects

This was a single-center, cross-sectional study. We consecutively enrolled 413 Japanese patients with T2D from our inpatients at the Diabetes Center of the Osaka City University Hospital who required hospitalization for glycemic control, education, and/or evaluation of diabetic complications between January 2009 and July 2014. The total sample size was estimated to be at least 360, so that as many as 12 explanatory factors could be included in a multiple regression analysis model consisting of two subgroups. T2D was diagnosed based on the criteria of American Diabetes Association [22] and Japan Diabetes Society [23]. Patients with type 1 diabetes or other types of diabetes were not included in this study. A smoker was defined as a current smoker or an ex-smoker. Preexisting CVDs, including coronary artery disease, cerebrovascular disease, or peripheral artery disease, were confirmed by medical records.

This study was performed in accordance with the Declaration of Helsinki (1975, as revised in 2013) and the Ethical Guidelines for Medical and Health Research Involving Human Subjects (The Japanese Ministry of Health, Labour and Welfare, 2014). The study protocol was approved by the ethics committee of the institution (Approval No. 3909). All study participants provided written informed consent.

### Physical and laboratory measurements

Blood pressure was determined using an automatic sphygmomanometer (Terumo Co., Ltd., Tokyo, Japan) with a conventional cuff after the subjects had rested for at least 5 min. Blood was drawn after an overnight fast and biochemical parameters were analyzed using a standard laboratory method at the Central Clinical Laboratory of the Osaka City University Hospital [16]. Glycated hemoglobin A1c (HbA1c) was assessed as the National Glycohemoglobin Standardization Program equivalent value (NGSP, %) according to the guidelines of Japan Diabetes Society [23]. The estimated glomerular filtration rate (eGFR) was calculated using the Japanese eGFR equation [24]. Immunoreactive insulin levels were measured for patients not receiving insulin therapy (*n* = 234) by electro-chemiluminescence immunoassay [Cobas 8000(502/602), Roche Diagnostics, K.K., Tokyo, Japan]. Homeostasis model assessment of insulin resistance (HOMA-R) was calculated according to the following formula: fasting insulin (μU/mL) × fasting glucose (mg/dL)/405 [16].

Fasting plasma levels of human omentin-1 (BioVendor, Candler, NC, USA) and human total adiponectin (Otsuka, Tokyo, Japan) were measured using an enzyme-linked immunosorbent assay following the manufacturer’s instructions. The intra- and interassay coefficients of variation for human omentin were < 5% and < 5%, respectively, whereas those of adiponectin were < 10% and < 10%, respectively.

### Measurements of carotid IMT with ultrasonography

The mean IMT of the common carotid artery (CCA) was measured by using a phase-locked echo-tracking system, which was equipped with a high-resolution real-time 13 MHz linear scanner (Prosound SSD 6500 and F75; Hitachi Aloka Medical, Ltd., Tokyo, Japan), as previously described [25]. The IMT value was determined by using a measurement software (Intimascope; Media Cross Co. Ltd, Tokyo, Japan), as described elsewhere [25, 26]. In brief, images were obtained 20 mm proximal to the origin of the bulb at the far wall of both CCAs. The average value of 416 points in this region and the largest value, including plaque lesions, in the CCA were measured separately. The mean-IMT of the right and left CCA (mean-IMT) was used as a marker of atherosclerotic change.

### Statistical analysis

Data were expressed as the number (%) or median [interquartile]. The subjects were divided into low-adiponectin group and high-adiponectin group according to the median value of plasma adiponectin (6.2 μg/mL). For comparisons between the groups, the χ^2^ test or Wilcoxon rank-sum test was performed as appropriate. Skewed parameters, such as triglycerides, HOMA-R, C-reactive protein, adiponectin levels, and omentin levels, were logarithmically transformed before regression analysis. A *p* value of < 0.20 was considered significant for interaction, as has been used in previous studies [16, 27], and a *p*-value of < 0.05 was considered significant for all other analyses. Statistical analyses were performed using the JMP 14 software program (SAS Institute Inc., Cary, NC, USA).

## Results

### Clinical characteristics, plasma omentin levels, and IMT

Clinical characteristics in all subjects and in subgroups of plasma adiponectin levels above and below the median are shown in Table 1. The median age, body mass index (BMI), and duration of diabetes of the total population were 65 years, 25.0 kg/m^2^, and 11 years, respectively. As expected, the high-adiponectin group showed lower BMI, immunoreactive insulin, HOMA-R, HbA1c, and triglycerides, higher high-density lipoprotein (HDL)-cholesterol, and C-reactive protein than the low-adiponectin group. Moreover, the high-adiponectin group exhibited older age, longer duration of diabetes, higher systolic blood pressure, higher serum creatinine, and lower eGFR than the low-adiponectin group. With respect to antidiabetic agents, high-adiponectin group showed higher prevalence of patients receiving thiazolidinediones and insulin, and lower one of patients receiving biguanides, than low-adiponectin group. No significant difference was found in the prevalence of preexisting CVDs, or the percentage of users of renin-angiotensin system (RAS) inhibitors or statins between the groups.

**Table 1:** Clinical characteristics, plasma omentin levels, and IMT in all subjects and in subgroups of plasma adiponectin levels above and below the median

	All subjects	Low-adiponectin (< 6.2 μg/mL)	High-adiponectin (≥ 6.2 μg/mL)	*p*
*N* (male, %)	413 (58.1)	206 (62.0)	207 (54.6)	0.146
Age (year)	65 [57–71]	63 [53–68]	67 [60–73]	< 0.001
Duration of diabetes (year)	11 [5–20]	9 [2–17]	15 [7–21]	< 0.001
BMI (kg/m^2^)	25.0 [22.1–27.9]	25.7 [23.1–28.5]	23.9 [21.1–27.1]	< 0.001
Systolic blood pressure (mmHg)	128 [116–143]	124 [113–136]	133 [118–148]	< 0.001
Diastolic blood pressure (mmHg)	74 [67–81]	74 [67–80]	73 [66–81]	0.760
Cardiovascular diseases (*n*, %)	86 (20.8)	42 (20.4)	44 (21.3)	0.828
Smoker (*n*, %)	198 (47.9)	109 (52.9)	89 (43.0)	0.044
Antihyperglycemic agents (*n*, %)				
None	44 (10.7)	24 (11.7)	20 (9.7)	0.512
Sulfonylureas	150 (36.3)	74 (35.9)	76 (36.7)	0.867
Biguanides	128 (31.0)	83 (40.3)	45 (21.7)	< 0.001
Dipeptidyl peptidase-4 inhibitors	125 (30.3)	63 (30.6)	62 (30.0)	0.889
Thiazolidinediones	44 (10.7)	10 (4.9)	34 (16.4)	< 0.001
GLP-1 receptor agonists	12 (2.9)	8 (3.9)	4 (1.9)	0.233
Insulin ± oral hypoglycemic agents	179 (43.3)	66 (32.0)	113 (54.6)	< 0.001
RAS inhibitors (*n*, %)	166 (40.2)	81 (39.4)	85 (41.1)	0.718
Statins (*n*, %)	176 (42.6)	88 (42.7)	88 (42.5)	0.966
Creatinine (mg/dL)	0.81 [0.66–1.06]	0.80 [0.65–0.96]	0.86 [0.67–1.27]	0.003
eGFR (mL/min/1.73 m^2^)	66.7 [51.9–78.8]	71.4 [57.7–82.6]	62.1 [42.3–74.6]	< 0.001
Fasting glucose (mg/dL)	119 [101–143]	121 [103–143]	117 [98–145]	0.511
HbA1c (%)	8.3 [7.3–9.6]	8.6 [7.5–9.7]	8.1 [7.0–9.6]	0.005
Immunoreactive insulin (μU/mL)^a^	6.9 [4.6–10.0]	7.7 [5.3–11.2]	5.7 [3.6–8.4]	< 0.001
HOMA-R^a^	2.03 [1.34–2.97]	2.29 [1.51–3.38]	1.59 [0.98–2.51]	< 0.001
Triglycerides (mg/dL)	116 [90–153]	127 [96–168]	108 [80–143]	0.001
Total-cholesterol (mg/dL)	176 [150–206]	170 [145–197]	180 [152–209]	0.037
HDL-cholesterol (mg/dL)	41 [36–50]	38 [34–44]	45 [38–56]	< 0.001
Non-HDL-cholesterol (mg/dL)	129 [106–161]	128 [105–162]	132 [106–160]	0.847
LDL-cholesterol (mg/dL)	105 [83–132]	102 [80–131]	110 [86–133]	0.121
C-reactive protein (mg/dL)^b^	0.07 [0.03–0.18]	0.10 [0.04–0.21]	0.05 [0.03–0.14]	0.010
Plasma adiponectin (μg/mL)	6.2 [3.8–11.0]	3.8 [2.7–4.9]	11.0 [7.9–15.9]	< 0.001
Plasma omentin (ng/mL)	573 [450–759]	510 [413–668]	630 [507–832]	< 0.001
IMT (mm)	0.81 [0.70–0.93]	0.79 [0.68–0.93]	0.82 [0.73–0.94]	0.064

Data are median [interquartile range] or *n* (%). *p*-values by χ^2^-test or Wilcoxon rank-sum test for comparison between the low-adiponectin group and high-adiponectin group

*IMT* intima-media thickness, *BMI* body mass index, *GLP-1* glucagon-like peptide-1, *RAS* renin-angiotensin system, *eGFR* estimated glomerular filtration rate, *HbA1c* glycated hemoglobin A1c, *HOMA-R* homeostasis model assessment of insulin resistance, *HDL* high-density lipoprotein, *LDL* low-density lipoprotein

^a^N = 234 for all subjects, n = 140 for low-adiponectin group, and n = 94 for high-adiponectin group not receiving insulin therapy

^b^N = 247 for all subjects, n = 132 for low-adiponectin group, and n = 115 for high-adiponectin group in whom serum C-reactive protein level was available

**Fig. 1:**
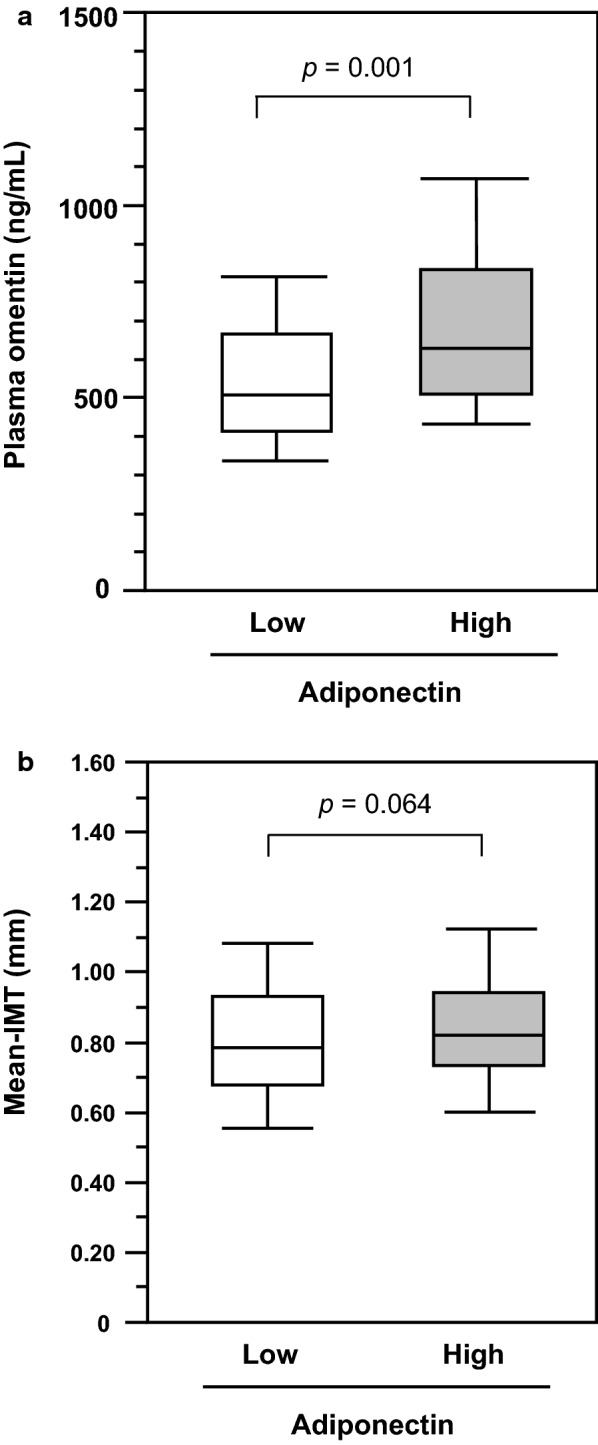
Comparison of plasma omentin levels (**a**) or mean IMT (**b**) between low- and high-adiponectin groups among patients with type 2 diabetes. Horizontal bars represent the 10th, 25th, 50th, 75th, and 90th percentile levels. *p*-values by Wilcoxon rank-sum test

The medians of plasma omentin levels, plasma adiponectin levels, and mean IMT in the total population were 573 ng/mL, 6.2 µg/mL, and 0.81 mm, respectively. Plasma omentin levels were higher, while the IMT tended to be greater in high-adiponectin group than low-adiponectin group (Table 1, Fig. 1).

### Correlations between plasma omentin levels, adiponectin levels, and CVD risk factors

We first examined the unadjusted correlations between plasma omentin levels, plasma adiponectin levels, and CVD risk factors. Plasma omentin levels were positively correlated with age (*ρ *= 0.421, *p* < 0.001), systolic blood pressure (*ρ *= 0.148, *p* = 0.003), serum creatinine (*ρ *= 0.243, *p* < 0.001), HDL-cholesterol (*ρ *= 0.231, *p* < 0.001), and adiponectin (*ρ *= 0.409, *p* < 0.001), and negatively correlated with BMI (*ρ *= − 0.318, *p* < 0.001), diastolic blood pressure (*ρ *= − 0.136, *p* = 0.006), eGFR (*ρ *= − 0.386, *p* < 0.001), immunoreactive insulin (*ρ *= − 0.197, *p* = 0.002), HOMA-R (*ρ *= − 0.171, *p* = 0.009), and C-reactive protein (*ρ *= − 0.234, *p* < 0.001). The associations of plasma adiponectin levels with these parameters were similar to those of omentin levels (data not shown). Indeed, the correlation between omentin levels and adiponectin levels was positive and independent of possible confounders (Table 2). Multivariate analyses also showed that both omentin and adiponectin levels were independently associated with lower eGFR and higher HDL-cholesterol. In addition, unlike adiponectin, omentin levels were independently associated with older age, lower BMI and higher HbA1c level in patients with T2D (Table 2).

**Table 2:** Correlations between plasma omentin levels or adiponectin levels and CVD risk factors

	Omentin		Adiponectin	
	*β*	*p*	*β*	*p*
Age	0.182	< 0.001	0.059	0.272
Sex (male = 1, female = 0)	− 0.067	0.111	− 0.003	0.946
BMI	− 0.168	< 0.001	− 0.007	0.888
Systolic blood pressure	0.039	0.372	0.108	0.015
eGFR	− 0.185	< 0.001	− 0.182	< 0.001
HbA1c	0.141	0.001	− 0.031	0.480
Log [triglycerides]	0.066	0.191	− 0.166	0.001
HDL-cholesterol	0.141	0.005	0.228	< 0.001
LDL-cholesterol	− 0.058	0.204	0.192	< 0.001
Log [adiponectin]	0.248	< 0.001	–	–
Log [omentin]	–	–	0.252	< 0.001
*R*^*2*^ *(p)*	0.353 (< 0.001)		0.342 (< 0.001)	

*β*, standard coefficient by multiple regression analysis. *R*^*2*^, coefficient of determination. Abbreviations are as in Table 1

### Multivariate analysis of the factors associated with IMT

To explore the association between plasma omentin levels and IMT, multiple regression analysis was performed with adjustment for potential confounders and adiponectin levels (Table 3). Neither omentin levels nor adiponectin levels were found to be independently associated with IMT in the total population. Then, an interaction analysis was performed to assess whether adiponectin levels modify the relationship between plasma omentin levels and IMT. The interaction analysis revealed a potential effect modification by adiponectin levels on the relationship between omentin levels and IMT (*p* for interaction = 0.087).

**Table 3:** Multiple regression analysis for the determinants of IMT

	All subjects		Low-adiponectin		High-adiponectin	
	*β*	*p*	*β*	*p*	*β*	*p*
Age	0.401	< 0.001	0.388	< 0.001	0.397	< 0.001
Sex (male = 1, female = 0)	0.063	0.217	0.099	0.163	0.010	0.898
BMI	− 0.133	0.010	− 0.048	0.514	− 0.214	0.003
Systolic blood pressure	0.121	0.015	0.104	0.121	0.147	0.046
eGFR	0.014	0.803	0.062	0.437	− 0.009	0.912
HbA1c	0.008	0.869	0.010	0.881	0.011	0.877
Non-HDL-cholesterol	0.093	0.060	0.152	0.032	0.055	0.439
Smoker (yes = 1, no = 0)	0.042	0.401	0.021	0.755	0.070	0.364
RAS inhibitor (yes = 1, no = 0)	− 0.016	0.719	− 0.023	0.734	− 0.008	0.909
Statin (yes = 1, no = 0)	− 0.025	0.592	0.051	0.454	− 0.091	0.171
Log [adiponectin]	− 0.015	0.761	0.035	0.614	0.008	0.902
Log [omentin]	− 0.055	0.313	0.067	0.379	− 0.170	0.022
*R*^*2*^ *(p)*	0.210 (< 0.001)		0.212 (< 0.001)		0.243 (< 0.001)	

*β*, standard coefficient by multiple regression analysis. *R*^*2*^, coefficient of determination. Abbreviations are as in Table 1

Therefore, we examined the association between omentin levels and IMT in low-adiponectin group and high-adiponectin group, separately (Table 3). The subgroup analysis revealed that plasma omentin levels were significantly and negatively associated with IMT in high-adiponectin group (*β* = − 0.170, *p* = 0.022), independently of adiponectin levels, sex, smoking habit, medications, and traditional CVD risk factors, but not in low-adiponectin group. In contrast to omentin levels, adiponectin levels were not significantly associated with IMT in either group (Table 3).

Additionally, HOMA-R was not found to be a significant determinant of IMT in all subjects, low-adiponectin group, or high-adiponectin group in participants not receiving insulin therapy (*n* = 234), although the relationship between IMT and HOMA-R or omentin levels were inconclusive due to insufficient sample size in high adiponectin group (Additional file 1: Table S1). We also performed a subgroup analysis of HbA1c stratified by the median (8.3%) to explore a potential implication of recent glycemic control in the link between omentin and IMT. The subgroup analysis demonstrated that HbA1c level was not associated with IMT in either group of HbA1c, and indicated that the relationship between omentin and IMT was not modified by HbA1c (*p* for interaction = 0.297) (Additional file 1: Table S2).

We also assessed the involvement of inflammation in IMT in the limited subjects for whom serum C-reactive protein levels were available (*n* = 247). Serum C-reactive protein levels were higher in low-adiponectin group than in high-adiponectin group (Table 1). Multivariate analysis showed that log [C-reactive protein] was an independent determinant of IMT in all subjects (Additional file 1: Table S3). In a subgroup analysis, log [C-reactive protein] was positively associated with IMT in low-adiponectin group, however, the association was not found in high-adiponectin group in which omentin level was inversely associated with IMT.

## Discussion

In the present study, we investigated the clinical features of T2D patients showing increased plasma adiponectin levels and the relationship between plasma omentin levels and carotid IMT in those patients. Our results demonstrated that the high-adiponectin group had increased omentin levels, multiple CVD risk factors, and non-significantly greater IMT, as compared to the low-adiponectin group of T2D patients. Moreover, in the high-adiponectin group, plasma omentin levels were found to be inversely associated with IMT, independently of adiponectin levels and traditional CVD risk factors, but not in the low-adiponectin group. To our knowledge, this study is the first to demonstrate the association between omentin and IMT which is modified by adiponectin level in patients with T2D.

This study first demonstrated that T2D patients with higher adiponectin levels (6.2 μg/mL or greater) had older age, higher systolic blood pressure, lower eGFR, as well as lower BMI, HbA1c, insulin resistance indices, and C-reactive protein, than those with lower adiponectin levels. The finding indicates the presence of multiple CVD risk factors in high-adiponectin group of T2D patients. A clustering of CVD risk factors in high-adiponectin group was not unexpected because previous studies have consistently associated higher adiponectin levels with older age [28] and chronic kidney disease [29]. Higher systolic blood pressure may also be explained by older age and lower eGFR in high-adiponectin group than in low-adiponectin group. Although inverse association between adiponectin and blood pressure is evidently shown in the general population [30], the adiponectin levels in hypertensive patients could be elevated depending on kidney function, as previously reported [31].

In addition to the clustering of CVD risk factors, carotid IMT tended to be greater in high-adiponectin group than low-adiponectin group. The finding contradicts the anti-atherogenic and anti-inflammatory properties of adiponectin [8]. However, recent cross-sectional studies have indicated that an inverse association between adiponectin level and IMT was significant only in healthy subjects or the general population, but not in patients with chronic disorders such as diabetes and inflammatory diseases [21]. Moreover, recent prospective studies suggest a positive, rather than the expected negative, relationship between adiponectin levels and cardiovascular and all-cause mortality in many clinical settings including type 2 diabetes [19, 20]. Several explanations have been proposed for the paradoxical relationship between adiponectin and mortality, including inflammation, weight loss, and the confounding role of natriuretic peptides [19, 20]. The inverse relationship between BMI and IMT only in high-adiponectin group (Table 3), which is contrary to what is generally shown in obese or pre-diabetic population [32], could also be understood in the context of paradoxical relationship between increased adiponectin levels and advanced atherosclerotic CVDs [19, 20]. Because plasma adiponectin level was not an independent determinant of IMT (Table 3), our data indicate that some factors related to older age and reduced BMI, rather than adiponectin itself, are implicated in the advanced atherosclerosis in high-adiponectin group.

This study clearly demonstrated an inverse relationship between omentin and IMT in T2D patients showing increased plasma adiponectin levels and multiple CVD risk factors. The inverse association between omentin and IMT is in line with clinical [6, 10, 11, 13–15] and experimental [33–35] studies reported previously. A number of studies demonstrated that omentin levels were inversely associated with carotid atherosclerosis, as evaluated by IMT [13, 14], carotid stenosis [11], plaque presence [6], or plaque instability [15], in non-diabetic population [11, 13–15] and in patients with T2D [6]. Experimental studies also consistently indicated a protective effect of omentin on atherosclerosis in mice [33–35] through attenuating inflammatory macrophages [33, 35] and migration/proliferation of smooth muscle cells [34, 35]. Because no prior study has assessed the relationship between omentin and atherosclerosis by using IMT only in T2D patients, this study provides additional evidence supporting an anti-atherogenic effect of omentin on atherosclerosis in humans.

It should be mentioned that plasma omentin levels were higher in T2D patients with higher adiponectin levels. Because omentin levels are known to be inversely correlated to parameters of obesity and the metabolic syndrome [4, 13, 36, 37], plasma omentin levels could be elevated in association with lower BMI, lower insulin resistance index, and higher HDL-cholesterol levels in high-adiponectin group of our T2D patients. The higher omentin levels could also be related to older age and lower eGFR in high-adiponectin group because circulating omentin levels are known to be elevated in patients with advanced chronic kidney disease [38]. Indeed, in the multivariate analysis (Table 2), lower BMI, higher HDL-cholesterol, older age, and lower eGFR were independent determinants of plasma omentin levels. Moreover, our data clearly showed, for the first time to our knowledge, a positive association between omentin and adiponectin levels which is independent of potential confounders. The data may raise a possibility that adiponectin directly upregulates omentin levels in humans. However, no experimental evidence has not been available on the molecular mechanism through which adiponectin regulates omentin production or secretion in human adipose tissue, which needs to be investigated in future studies.

This study demonstrated for the first time that the relationship between omentin levels and IMT varies depending on plasma adiponectin levels. An inverse association between omentin and IMT was found in high-adiponectin group but not in low-adiponectin group. As discussed above, omentin levels were increased in association with several CVD risk factors and possibly with increased adiponectin levels in high-adiponectin group. While studies in individuals without CVDs indicated an inverse relationship between omentin and subclinical atherosclerosis [6, 11, 13, 14], studies in patients with established CVDs demonstrated that higher omentin levels were cross-sectionally associated with the severity of coronary artery disease [35, 39] and were longitudinally associated with adverse CVD outcome [40, 41]. Increased plasma omentin levels were also demonstrated after acute cardiac ischemia in patients undergoing cardiac surgery [42]. Importantly, in patients with coronary artery disease and ≥ 90% coronary occlusion, plasma omentin levels were not only increased with the disease severity, but also were independently associated with better collateral circulation, or better endothelial function [39]. Besides, we recently found that plasma omentin levels were increased and independently associated with better endothelial function in high CVD risk subgroups of patients with T2D, but not in low-risk patients [16]. Taken together, our data suggest that omentin levels are increased to compensate for vascular damage and/or atherogenesis due to accumulation of CVD risk factors in T2D patients with increased adiponectin levels.

Because both omentin and adiponectin are considered to be the adipokines which exert protective action against atherosclerosis [8], it is noteworthy that only omentin, but not adiponectin, showed a significant inverse association with atherosclerosis. It is evidently known that hyperglycemic status, or diabetes, affects the pro-inflammatory/oxidative properties and the pro-thrombotic properties in the arterial plaque lesion [43–45]. Evidence from experimental studies has established the anti-inflammatory, anti-oxidative, and anti-thrombotic properties of adiponectin [8]. However, recent meta-analyses have indicated that a direction of the relationship between circulating adiponectin level and IMT or carotid plaque presence is dependent on the severity of underlying disease, while increased adiponectin levels are associated with an increased risk of ischemic stroke [21, 46]. These reports could associate increased adiponectin levels with carotid plaque instability. A recent human study also demonstrated an overall abundance of adiponectin with a decreased adiponectin receptor 2 expression and activity in unstable plaques in patients which underwent a carotid endarterectomy [47], suggesting a failure of adiponectin action in unstable carotid plaque. Our results are in accordance with previous reports [21, 46] including ours [25] that failed to associate adiponectin levels with carotid IMT in patients with T2D and suggested a loss of anti-atherogenic effect of adiponectin in T2D. In contrast to adiponectin, the anti-atherogenic effect of omentin was evidently shown in our T2D patients even with elevated CVD risk. While both adipokines were correlated with lower eGFR, omentin levels, but not adiponectin levels, were also independently associated with older age and higher HbA1c levels (Table 2). Taken together, we speculate that omentin levels were upregulated by CVD risk factors, such as aging, renal dysfunction, and chronic hyperglycemia, more potently than adiponectin to play a role against atherosclerosis in patients with T2D.

It needs to be mentioned that insulin resistance [48] and/or hyperglycemic status [49] could also affect IMT in patients with T2D. Our data do not exclude the possible involvement of HOMA-R in the link between omentin and IMT, because the sample size was not enough in the high-adiponectin group. In our results, HbA1c levels were lower in high-adiponectin group than low-adiponectin group, suggesting that hyperglycemia is not involved in the advanced atherosclerosis in high-adiponectin group. Moreover, results from the subgroup analysis by HbA1c level suggest that glycemic control does not affect the relationship between omentin and IMT in T2D patients. Taken together with the results of Table 3, it is suggested that some factors related to older age and/or lower BMI, rather than hyperglycemia over the last few months, are implicated in the advanced atherosclerosis in high-adiponectin group.

A potential implication of inflammation in IMT in patients with T2D [32, 43] also needs to be considered. The additional data for the limited subjects suggest that inflammation as assessed by C-reactive protein is involved in atherosclerosis in low-adiponectin group, but not in high-adiponectin group (Additional file 1: Table S3). Considering that omentin levels were inversely associated with IMT in high-adiponectin group, as was observed in the full sample, it is speculated that increased omentin level in high-adiponectin group plays a role against atherosclerosis through its anti-inflammatory effect.

With respect to the anti-atherogenic effect of antidiabetic agents, 33.2% of the participants were receiving incretin-related drugs, i.e. dipeptidyl peptidase-4 inhibitors or glucagon-like peptide-1 receptor agonists, which were recently shown to have beneficial effects on CVD outcomes [50, 51] and on the progression of carotid IMT [52] in patients with T2D. We found that the IMT was lower in users of these drugs than in non-users (median IMT, 0.78 vs. 0.83 mm, *p* = 0.015). However, the use of incretin-related drugs was not an independent determinant of IMT after adjustment for potential confounders (*β* = − 0.078, *p* = 0.094). Because this study was not primarily designed to evaluate the effect of these drugs on IMT, our data never exclude the possible effect of incretin-related drugs on IMT in our study participants.

This study has several limitations. First, neither adipokines other than adiponectin nor proinflammatory cytokines were evaluated, with which the effect of omentin on atherosclerosis would have been characterized better than with adiponectin alone. Second, because over 40% of the participants were receiving insulin therapy, we could not fully assess the possible effect of HOMA-R on IMT. Third, this study did not utilize atherectomy specimens, with which we could apply histological and/or molecular approaches. Forth, we did not measure waist circumference or visceral fat area using computed tomography, which would be more closely correlated with adiponectin levels than BMI and could affect the results obtained. Fifth, the participants were receiving RAS inhibitors, statins, and/or anti-hyperglycemic agents including which could have affected omentin levels, adiponectin levels, IMT, and/or CVD risk factors. To minimize the effect of at least RAS inhibitors and statins on IMT, use of these drugs was adjusted for in multivariate analyses. Sixth, the main outcome of this study was IMT, which was shown to have limited value for predicting CVD outcomes [53] and is not recommended for assessing CVD risk in recent guideline [54]. Finally, because of the consecutive manner of subject inclusion in the university hospital, our participants with T2D had long disease duration, poor glycemic control, and high prevalence of macrovascular complications. Thus, the present results may not be generalized to the entire population of patients with T2D.

## Conclusions

This study demonstrated that plasma omentin levels are independently and inversely associated with IMT in patients with T2D and increased adiponectin levels, who have multiple CVD risk factors. Our data suggest a protective role of omentin on atherosclerosis in patients with T2D and increased adiponectin levels, who have characteristics of high CVD risk. This study further suggests that the effect of omentin on atherosclerosis is modified by circulating adiponectin levels. Further experimental studies are needed to determine a direct interaction between omentin and adiponectin in the context of atherosclerosis in T2D. Longitudinal studies are also warranted to confirm whether plasma omentin levels are predictive of the progression of atherosclerosis in T2D patients with increased plasma adiponectin levels, e.g., non-obese older adults or those with chronic kidney disease.

## Supplementary information

**Additional file 1.** Additional tables.

## Data Availability

The datasets used and analyzed during the current study are available from the corresponding author on reasonable request.

## Abbreviations

T2D: type 2 diabetes
IMT: intima-media thickness
CVD: cardiovascular disease
HbA1c: glycated hemoglobin A1c
NGSP: National Glycohemoglobin Standardization Program
eGFR: estimated glomerular filtration rate
HOMA-R: homeostasis model assessment of insulin resistance
CCA: common carotid artery
BMI: body mass index
HDL: high-density lipoprotein
LDL: low-density lipoprotein
RAS: renin-angiotensin system
GLP: glucagon-like peptide
