# Supplementary material for: Plasma omentin levels are inversely associated with atherosclerosis in type 2 diabetes patients with increased plasma adiponectin levels: a cross-sectional study
Source: Cardiovasc Diabetol. 2019 Dec 5;18:167. doi: 10.1186/s12933-019-0973-3 (PMC6894467; doi:10.1186/s12933-019-0973-3)
Supplement: Supplementary file 1 — Additional file 1. Additional tables. [file 12933_2019_973_MOESM1_ESM.docx]

**Additional file 1: Table S1. Multiple regression analysis for the determinants of IMT**

|  | **All subjects**  **(N = 234)** | | **Low-adiponectin**  **(< 6.2 μg/mL)**  **(N = 140)** | | **High-adiponectin**  **(****≥ 6.2 μg/mL)**  **(N = 94)** | |
| --- | --- | --- | --- | --- | --- | --- |
|  | ***β*** | ***p*** | ***β*** | ***p*** | ***β*** | ***p*** |
| Age | 0.491 | <0.001 | 0.488 | <0.001 | 0.405 | 0.005 |
| Sex (male = 1, female = 0) | 0.060 | 0.373 | 0.029 | 0.734 | 0.072 | 0.560 |
| BMI | –0.112 | 0.134 | –0.066 | 0.493 | –0.170 | 0.192 |
| Systolic blood pressure | 0.101 | 0.134 | 0.106 | 0.182 | 0.176 | 0.190 |
| eGFR | 0.118 | 0.119 | 0.093 | 0.305 | 0.080 | 0.560 |
| HbA1c | 0.017 | 0.793 | 0.028 | 0.729 | –0.015 | 0.898 |
| Non-HDL-cholesterol | 0.154 | 0.023 | 0.235 | 0.007 | 0.077 | 0.521 |
| Smoker (yes = 1, no = 0) | 0.064 | 0.332 | 0.086 | 0.297 | –0.013 | 0.917 |
| RAS inhibitor (yes = 1, no = 0) | 0.037 | 0.553 | 0.064 | 0.416 | –0.046 | 0.694 |
| Statin (yes = 1, no = 0) | 0.011 | 0.862 | 0.031 | 0.698 | 0.019 | 0.861 |
| Log [HOMA-R] | 0.122 | 0.090 | 0.099 | 0.270 | 0.110 | 0.397 |
| Log [adiponectin] | –0.010 | 0.890 | 0.089 | 0.305 | –0.068 | 0.561 |
| Log [omentin] | –0.004 | 0.954 | 0.019 | 0.840 | –0.076 | 0.540 |
| *R^2^ (p)* | 0.240 (<0.001) | | 0.292 (<0.001) | | 0.211 (0.090) | |

*β*, standard coefficient by multiple regression analysis. *R^2^*, coefficient of determination. Abbreviations are as in Table 1.

**Additional file 1: Table S2. Multiple regression analysis for IMT in subgroups stratified by the median HbA1c**

|  | **All subjects**  **(N = 413)** | | **Low-HbA1c**  **(< 8.3%)**  **(N = 207)** | | **High-HbA1c**  **(≥ 8.3%)**  **(N = 206)** | |
| --- | --- | --- | --- | --- | --- | --- |
|  | ***β*** | ***p*** | ***β*** | ***p*** | ***β*** | ***p*** |
| Age | 0.401 | <0.001 | 0.406 | <0.001 | 0.354 | <0.001 |
| Sex (male = 1, female = 0) | 0.063 | 0.217 | 0.147 | 0.040 | –0.040 | 0.586 |
| BMI | –0.133 | 0.010 | –0.145 | 0.042 | –0.096 | 0.208 |
| Systolic blood pressure | 0.121 | 0.015 | 0.175 | 0.011 | 0.050 | 0.500 |
| eGFR | 0.014 | 0.803 | 0.109 | 0.198 | –0.068 | 0.383 |
| HbA1c | 0.008 | 0.869 | 0.080 | 0.220 | –0.075 | 0.294 |
| Non-HDL-cholesterol | 0.093 | 0.060 | 0.132 | 0.042 | 0.095 | 0.225 |
| Smoker (yes = 1, no = 0) | 0.042 | 0.401 | 0.004 | 0.959 | 0.080 | 0.272 |
| RAS inhibitor (yes = 1, no = 0) | –0.016 | 0.719 | 0.092 | 0.150 | –0.146 | 0.037 |
| Statin (yes = 1, no = 0) | –0.025 | 0.592 | –0.033 | 0.606 | 0.016 | 0.813 |
| Log [adiponectin] | –0.015 | 0.761 | 0.015 | 0.840 | 0.025 | 0.736 |
| Log [omentin] | –0.055 | 0.313 | –0.078 | 0.333 | –0.036 | 0.639 |
| *R^2^ (p)* | 0.210 (<0.001) | | 0.274 (<0.001) | | 0.200 (<0.001) | |

*β*, standard coefficient by multiple regression analysis. *R^2^*, coefficient of determination. Abbreviations are as in Table 1.

**Additional file 1: Table S3. Multiple regression analysis for the determinants of IMT**

|  | **All subjects**  **(N = 247)** | | **Low-adiponectin**  **(< 6.2 μg/mL)**  **(N = 132)** | | **High-adiponectin**  **(≥ 6.2 μg/mL)**  **(N = 115)** | |
| --- | --- | --- | --- | --- | --- | --- |
|  | ***β*** | ***p*** | ***β*** | ***p*** | ***β*** | ***p*** |
| Age | 0.427 | <0.001 | 0.424 | <0.001 | 0.403 | <0.001 |
| Sex (male = 1, female = 0) | 0.073 | 0.269 | 0.068 | 0.459 | 0.020 | 0.851 |
| BMI | –0.238 | 0.001 | –0.141 | 0.136 | –0.288 | 0.011 |
| Systolic blood pressure | 0.072 | 0.258 | 0.195 | 0.020 | –0.034 | 0.735 |
| eGFR | 0.024 | 0.751 | 0.134 | 0.196 | –0.092 | 0.410 |
| HbA1c | 0.063 | 0.327 | 0.119 | 0.171 | 0.089 | 0.403 |
| Non-HDL-cholesterol | 0.061 | 0.365 | 0.126 | 0.161 | 0.005 | 0.967 |
| Smoker (yes = 1, no = 0) | 0.068 | 0.295 | –0.023 | 0.790 | 0.178 | 0.096 |
| RAS inhibitor (yes = 1, no = 0) | –0.035 | 0.559 | –0.044 | 0.597 | 0.017 | 0.857 |
| Statin (yes = 1, no = 0) | –0.012 | 0.845 | 0.057 | 0.537 | –0.068 | 0.461 |
| Log [C-reactive protein] | 0.194 | 0.003 | 0.254 | 0.003 | 0.114 | 0.286 |
| Log [adiponectin] | 0.078 | 0.246 | 0.114 | 0.184 | 0.076 | 0.453 |
| Log [omentin] | –0.121 | 0.090 | 0.003 | 0.971 | –0.228 | 0.028 |
| *R^2^ (p)* | 0.262 (<0.001) | | 0.318 (<0.001) | | 0.301 (<0.001) | |

*β*, standard coefficient by multiple regression analysis. *R^2^*, coefficient of determination. Abbreviations are as in Table 1.
